# Neuropilin-1: A Conserved Entry Receptor for SARS-CoV-2 and a Potential Therapeutic Target

**DOI:** 10.3390/biomedicines13071730

**Published:** 2025-07-15

**Authors:** Vivany Maydel Sierra-Sánchez, Citlali Margarita Blancas-Napoles, Aina Daniela Sánchez-Maldonado, Indira Medina, Rodrigo Romero-Nava, Fengyang Huang, Enrique Hong, Asdrúbal Aguilera-Méndez, Sergio Adrian Ocampo-Ortega, Santiago Villafaña

**Affiliations:** 1 Laboratorio de Terapia Génica Experimental, Escuela Superior de Medicina, Instituto Politécnico Nacional, Ciudad de México 11340, Mexico; vivany.s44@gmail.com (V.M.S.-S.); citla91@hotmail.es (C.M.B.-N.); aina.sanchez81@gmail.com (A.D.S.-M.); imedinar2400@alumno.ipn.mx (I.M.); roloromer@gmail.com (R.R.-N.); 2 Laboratorio de Investigación en Obesidad y Asma, Hospital Infantil de México “Federico Gómez”, Ciudad de Mexico 06720, Mexico; f_y_huang@yahoo.com; 3 Departamento de Farmacobiología, Centro de Investigación y de Estudios Avanzados, Ciudad de Mexico 14330, Mexico; ehong@gmail.com; 4 Instituto de Investigaciones Químico Biológicas, Universidad Michoacana de San Nicolás Hidalgo, Morelia 58040, Mexico; amendez@umich.mx

**Keywords:** NRP1, ACE2, differential expression analysis, RRAR domain, genetic conservation, gene expression, viral receptor, siRNA

## Abstract

**Background/Objectives**: Neuropilin-1 (NRP1) is a key co-receptor for SARS-CoV-2, complementing the ACE2 receptor. Several investigations have documented highly conserved sequences in this receptor, supporting the implication of NRP1 as a key mediator in SARS-CoV-2 cellular entry mechanisms. **Methods**: To investigate this hypothesis, we examined 104,737 SARS-CoV-2 genome fastas from GISAID genomic data, corresponding to isolates collected between 2020 and 2025 in Mexico. Specifically, we focused on the RRAR motif, a known furin-binding site for NRP-1 and the binding site for ACE2 with the spike protein. Our analysis revealed high conservation (>98%) of the RRAR domain compared to a rapidly diminishing ACE2-binding domain. A complementary analysis, using Data from Gene Expression Omnibus (GEO, GSE150316), showed that NRP1 expression in lung tissue remains relatively stable, whereas ACE2 displayed high inter-individual variability and lower abundance compared to NRP1. Based on this evidence, we designed two humans–rats NRP1 siRNAs that were tested in vivo using a melittin-induced lung injury model. **Results**: The RT-PCR assays confirmed an effective NRP1 knockdown, and the siRNA-treated group showed a significant reduction in the lesions severity. These findings highlight *NRP1* as a stable and relevant therapeutic target and suggest the protective potential of siRNA-mediated gene silencing. **Conclusions**: The evidence presented here supports the rational design of NRP1-directed therapies for multiple circulating SARS-CoV-2 variants in Mexico.

## 1. Introduction

Neuropilin-1 (NRP1) is a multifunctional transmembrane receptor initially characterized for its involvement in axonal guidance, angiogenesis, and immunoregulatory processes. More recently, it has gained attention as an auxiliary receptor that facilitates the cellular entry of SARS-CoV-2. This role is mediated through a critical post-binding event: following the initial interaction between the viral spike (S) glycoprotein and its primary receptor, angiotensin-converting enzyme 2 (ACE2), the host protease furin—widely expressed in human tissues—cleaves the spike protein at the S1/S2 boundary. This cleavage exposes a polybasic C-terminal motif encompassing residues 682–685 (RRAR), which conforms to the canonical C-end Rule (CendR) motif, defined by the consensus sequence R/KXXR/K. This specific arrangement promotes binding to the b1 domain of NRP1. In the case of SARS-CoV-2, the exposed RRAR sequence after furin cleavage fits this pattern exactly: it has arginines at positions 1 and 4, and alanine and another arginine at positions 2 and 3, respectively—thus satisfying the RXXR configuration. The presence of this motif is essential for NRP1 recognition, as it mimics physiological ligands that naturally interact with this receptor via their C-terminal CendR sequences [1].

The exposure of the RRAR motif enables high-affinity binding to the b1 domain of NRP1, a region known for recognizing and internalizing CendR-containing ligands. Structural, biochemical, and molecular dynamics studies have demonstrated that this interaction significantly enhances viral entry via endocytic pathways, particularly in cell types that exhibit low ACE2 expression. Specifically, crystallographic data reveal that the terminal arginine residue (R685) of the spike protein forms stable electrostatic and cation–π interactions with conserved aromatic residues within NRP1’s b1 domain—namely Y297, W301, and Y353. These findings underscore the importance of NRP1 not only as a facilitator of viral internalization, but also as a determinant of expanded viral tropism beyond tissues that primarily express ACE2 [2,3,4].

Functional and computational mutagenesis analyses have demonstrated that deletion of the RRAR motif or replacement of the terminal arginine residue (R685) severely disrupts the spike–NRP1 interaction, leading to reduced viral infectivity. Notably, Liu et al. (2021) employed molecular dynamics simulations and binding free energy calculations to confirm that both cation–π and salt bridge interactions are critical for maintaining a stable spike–NRP1 complex [5,6].

Although NRP1 does not mediate the initial attachment of SARS-CoV-2 to the cell surface—a process primarily governed by the ACE2 receptor—it plays a critical downstream role in promoting viral internalization via an endocytic mechanism that closely resembles macropinocytosis. Experimental evidence demonstrated that genetic depletion of NRP1 through shRNA or its pharmacological inhibition significantly reduced intracellular viral RNA levels [3], inhibited syncytia formation, and curtailed overall viral propagation in neuronal cells. These findings emphasize the functional importance of the S1 CendR–NRP1 axis as a co-receptor pathway that enhances SARS-CoV-2 infectivity, particularly in tissues with low ACE2 expression [2,3]

However, the virus is prone to mutation, which complicates the development of long-lasting therapeutic options. Although a vaccine against SARS-CoV-2 currently exists, it requires periodic reformulation due to the emergence of dominant viral variants. Therefore, identifying therapeutic strategies that target highly conserved viral elements—less prone to frequent mutation—is of critical importance. Based on this rationale, we propose NRP1 as a viable therapeutic target, owing to the genetic conservation and stability of its viral interaction site [7,8].

This study evaluates the efficacy of neuropilin-1 (NRP1) gene silencing through the application of small interfering RNAs (siRNAs), with the aim of exploring its therapeutic potential in the context of SARS-CoV-2 infection. Specifically, the research (1) analyzes the conservation of the RRAR motif among circulating Mexican variants of SARS-CoV-2, using genomic sequences retrieved from the GISAID database, to confirm NRP1 as a stable viral entry receptor; (2) compares pulmonary gene expression profiles of NRP1 and ACE2 in healthy individuals and COVID-19 patients, using transcriptomic datasets obtained from the Gene Expression Omnibus (GEO); and (3) assesses the in vivo efficacy of siRNA-mediated NRP1 knockdown in mitigating lung injury induced by melittin in a Wistar rat model.

## 2. Materials and Methods

### 2.1. Conservation Analysis of the SARS-CoV-2 Viral Binding Domain to NRP1 in Mexican Variants Using Bioinformatics Tools

To evaluate the relevance of NRP1 as a therapeutic target, a bioinformatic analysis was performed using SARS-CoV-2 genomic sequences isolated in Mexico between 2020 and March 2025, retrieved from the GISAID database (https://www.gisaid.org/), additionally, data reported by the Mexican government (https://datos.covid-19.conacyt.mx/#DownZCSV (accessed on 4 July 2025)) were used to calculate the case fatality rate. The analysis specifically focused on the region encoding the RRAR motif (Arg-Arg-Ala-Arg), which mediates the interaction between the virus and the NRP1 co-receptor. In the Wuhan reference sequence, this motif corresponds to the nucleotide fragment CGG-CGG-GCA-CGT.

Multiple sequence alignments were conducted using a custom-made software developed in Python (Python Software Foundation, version 3.13.2, Wilmington, DE, USA) by our laboratory, designed to perform multiple sequence alignments of viral FASTA files. This internal tool was used to identify the presence and conservation of the RRAR motif within the circulating SARS-CoV-2 variants in Mexico.

### 2.2. Conservation Analysis of the SARS-CoV-2–ACE2 Interaction Site in Mexican Variants Using Bioinformatics Tools

Similarly, to assess the relevance of the ACE2-binding site as a potential therapeutic target, a bioinformatic analysis was performed using SARS-CoV-2 genomic sequences isolated in Mexico between 2020 and March 2025 from the GISAID database (https://www.gisaid.org/).

This analysis focused on the region encoding key residues involved in the interaction between the viral spike protein and the ACE2 receptor, particularly L455, F486, Q493, S494, and N501.

The corresponding nucleotide sequence:

TTG TTT AGG AAG TCT AAT CTC AAA CCT TTT GAG AGA GAT ATT TCA ACT GAA ATC TAT CAG GCC GGT AGC ACA CCT TGT AAT GGT GTT GAA GGT TTT AAT TGT TAC TTT CCT TTA CAA TCA TAT GGT TTC CAA CCC ACT AAT

was used to perform conservation analyses across viral genomes.

### 2.3. Gene Expression Analysis of NRP1 and ACE2 in Human Lung Tissue Using Publicly Available Data

The transcriptomic dataset used to compare pulmonary gene expression of NRP1 and ACE2 was obtained from the Gene Expression Omnibus (GEO) (National Center for Biotechnology Information, Bethesda, MD, USA; https://www.ncbi.nlm.nih.gov/geo/ (accessed on 4 July 2025)) under accession number GSE150316. This dataset included 11 samples, comprising 5 healthy control lung tissues (samples GSM4546608–GSM4546612) and 6 lung tissues from deceased COVID-19 patients (samples GSM4546576, GSM4546596, GSM4698526, GSM4698536, GSM4698544, and GSM4698549).

Available metadata for these samples included sex, age range, and disease status. The control group consisted of 3 males and 2 females with an estimated mean age of 55 years. The COVID-19 group included 4 males and 2 females with an estimated mean age of 58 years. Disease severity was uniformly defined as fatal COVID-19 based on post-mortem sampling. Although we verified that all samples came from the same study, sequencing batch, and tissue type to minimize technical confounding.

To evaluate differential expression between groups, we considered as biologically relevant those genes showing a log_2_ fold change (log_2_FC) ≥ ±2.0 and an associated adjusted *p*-value (FDR) < 0.05, following Benjamini–Hochberg correction.

To explore differences in expression between NRP1 and ACE2 across both sample groups, we first visualized data using boxplots in RStudio RStudio v4.2 (Posit Software, Boston, MA, USA). Statistical comparisons were conducted using paired Wilcoxon signed-rank tests to compare the expression levels of NRP1 and ACE2 within the same samples.

### 2.4. Design and Synthesis of siRNA Targeting NRP1

Human and rat NRP1 gene variants were identified through the NCBI GeneBank, and their respective coding sequences (CDS) were selected.

The coding regions were aligned using the Clustal Omega (EMBL-EBI, Hinxton, Cambridgeshire, UK; https://www.ebi.ac.uk/Tools/msa/clustalo/ (accessed on 4 July 2025)) server to identify conserved regions between species.

Candidate siRNA sequences were designed using the siRNA Wizard v3.1 siRNA Wizard v3.1 (InvivoGen, San Diego, CA, USA; https://www.invivogen.com/sirnawizard/design.php (accessed on 4 July 2025)), based on the following criteria:

- Sequence length 21 nucleotides.
- GC content 45–55%.
- Targeting the coding sequence (CDS).
- Absence of off-target homology with unrelated genes.

Selected siRNA sequences were then mapped onto the secondary structure of NRP1 mRNA using RNAfold web server (Institute for Theoretical Chemistry, University of Vienna, Vienna, Austria; http://rna.tbi.univie.ac.at/ (accessed on 4 July 2025)). to ensure their hybridization occurred in accessible loop regions, enhancing silencing efficiency. The synthesis of siRNAs was carried out using the Mermade 8 synthesizer (BioAutomation, Plano, TX, USA), following a four-step process:

1. Detritylation of the 5′ terminus to expose reactive groups;
2. Coupling of phosphoramidites to elongate the oligonucleotide in the 3′ to 5′ direction;
3. Capping of unreacted hydroxyl groups to prevent truncation products;
4. Oxidation to stabilize the phosphodiester bonds.

Standard CPG supports with a pre-attached nucleoside were used.

Post-synthesis, the siRNAs were cleaved from the solid support using ammonium hydroxide, deprotected at the 2′ position using triethylamine trihydrofluoride, and purified precipitated with butanol, and subsequently washed with ethanol to remove residual impurities. Final siRNA products were resuspended in nuclease-free water and quantified by absorbance at 260 nm using an Implen^®^ nanophotometer (Implen GmbH, Munich, Germany). The concentration and purity were verified, ensuring optimal conditions for subsequent biological evaluation.

### 2.5. In Vivo Model and Functional Evaluation of Both siRNAs

A total of 24 male Wistar rats (300–350 g) were used in the study. Animals were maintained under controlled environmental conditions with a 12/12-h light–dark cycle and had free access to food and water. All experimental procedures were approved by the Institutional Animal Care and Use Committee of the Escuela Superior de Medicina, Instituto Politécnico Nacional, and were conducted in accordance with the Mexican Official Standard NOM-062-ZOO-1999 [9] for the care and use of laboratory animals.

Animals were randomly assigned to the experimental groups (*n* = 5 per group) using a simple randomization method based on random number generation. The individual animal was used as the unit of randomization. Group allocation was performed prior to the acclimatization period to ensure unbiased distribution across all groups.

Only healthy animals within the specified weight range and without visible signs of illness or abnormal behavior were included. Animals were acclimated for one week prior to the start of the experimental procedures. No animals were excluded from the study after group allocation, and all 24 animals completed the protocol and were included in the final analysis. Blinding was not implemented due to the exploratory nature of the study and because outcome measures were based on objective, quantifiable data (RT-qPCR and MTT assays), minimizing the risk of observer bias.

All procedures involving animals were conducted under deep anesthesia to minimize pain and distress. Anesthetic induction was achieved using intraperitoneal administration of ketamine (75 mg/kg) and xylazine (10 mg/kg). Animals were monitored throughout the procedure for signs of discomfort or inadequate anesthesia, including respiratory rate and pedal withdrawal reflex. During the experimental phase, care was taken to ensure minimal handling and reduced environmental stress. At the end of the protocol, animals were humanely euthanized with an overdose of sodium pentobarbital (120–200 mg/kg, intraperitoneal), in accordance with institutional ethical guidelines. No additional analgesics were administered due to the acute nature of the model and the short duration of procedures under full anesthesia.

Animals were randomly assigned into four experimental groups (*n* = 5 per group) as follows:

1. Control group (*n* = 5); no surgical intervention;
2. Sham group (*n* = 5); surgical procedure without administration of vehicle or siRNA;
3. Vehicle group (*n* = 5); administration of vehicle only;
4. siRNA-treated group (*n* = 5); administration of designed siRNAs.

siRNA administration was performed via the jugular vein, using a mixture containing 50 µL of siRNAs, 400 µL of glucose solution, and 6 µL of Turbofect^®^ (Thermo Fisher Scientific, Waltham, MA, USA) as a transfection agent.

Seventy-two hours after treatment, animals were euthanized by intraperitoneal injection of sodium pentobarbital (200 mg/kg), and lung tissues were collected for subsequent analyses.

### 2.6. Evaluation of NRP-1 Expression by Quantitative RT-PCR

Total RNA was extracted from lung tissue using TriPure Isolation Reagent^®^ (Roche Diagnostics, Mannheim, Germany), and RNA purity was assessed by measuring absorbance ratios at 260/280 nm and 260/230 nm.

Complementary DNA (cDNA) synthesis was performed using the ImProm-II™ Reverse Transcription System (Promega Corporation, Madison, WI, USA) following the thermal protocol: 25 °C for 5 min, 42 °C for 60 min, and 70 °C for 15 min.

Quantitative PCR (qPCR) was carried out using SYBR Green^®^ (Thermo Fisher Scientific, Waltham, MA, USA) chemistry on a LightCycler^®^ Nano System (Roche Diagnostics, Mannheim, Germany).

Primers specific for NRP-1 and reference genes were designed with the Primer-BLAST (National Center for Biotechnology Information, Bethesda, MD, USA; https://www.ncbi.nlm.nih.gov/tools/primer-blast/ (accessed on 4 July 2025)) and synthesized by T4 Oligo (T4 Oligo, Irapuato, Guanajuato, Mexico).

The qPCR amplification program consisted of:

- Initial denaturation: 50 °C for 2 min, followed by 95 °C for 2 min;
- Amplification cycles: 45 cycles of 95 °C for 15 s and 60 °C for 30 s.

All samples were analyzed in triplicate, and relative gene expression was calculated using the 2^−ΔCt^ method, normalizing against the housekeeping genes β-actin (ACTB) and HPRT1.

### 2.7. Evaluation of siRNA in a Melittin-Induced Lung Inflammation Model

An acute lung injury model was established in male Wistar rats by intratracheal administration of melittin at a dose of 0.17 mg/kg, dissolved in 0.9% saline solution. To evaluate the protective effect of NRP1 silencing, animals were randomly assigned to two experimental groups (*n* = 5 per group):

Control group: melittin only
Treatment group: melittin + siRNAs 1 and 2 targeting NRP1

In the treatment group, siRNA was administered intravenously via the jugular vein, in a total volume of 50 µL, using a formulation containing Turbofect^®^ as a transfection agent, 72 h prior to melittin administration. This interval was selected based on our gene expression data, which demonstrated optimal NRP1 knockdown 72 h post-injection.

Subsequently, melittin was administered 30 min before euthanasia, to induce acute lung injury at a time point when NRP1 expression was already suppressed. After 30 min, animals were euthanized, and lung tissues were collected for imaging analyses.

For quantification of tissue injury, digital images of the extracted lungs were captured and analyzed using ImageJ software (version 1.49, National Institutes of Health, Bethesda, MD, USA; https://imagej.net/ij/ (accessed on 4 July 2025)). The total lung area and lesion area were measured, and the percentage of affected tissue was calculated as an indicator of the extent of pulmonary damage induced by melittin. After lung extraction, macroscopic injury was quantified through standardized digital photographs and analyzed using ImageJ software. Images were first calibrated using a known scale bar to convert pixel-based measurements into square centimeters. The total lung area was determined by manually outlining the entire lung surface using the Freehand Selection Tool, and measuring the selection with the Analyze > Measure function. Lesion areas were identified based on visible discoloration—typically darkened or hemorrhagic zones—and were similarly delineated. In cases of multiple lesions, individual regions were added to the ROI Manager and then measured collectively. The percentage of affected lung area was calculated by dividing the lesion area by the total lung area and multiplying the result by 100.

### 2.8. Statistical Analysis

Gene expression data, reported as transcripts per million (TPM), were obtained from RNA sequencing (RNA-Seq) studies. Differential expression analysis was performed using the DESeq2 package (version 1.44.0, https://bioconductor.org/packages/release/bioc/html/DESeq2.html (accessed on 4 July 2025)) within the R statistical environment, allowing for data normalization and the identification of differentially expressed genes between experimental groups. Subsequently, the results were validated through one-way analysis of variance (ANOVA), followed by Tukey’s post hoc test for multiple comparisons. In addition, paired Wilcoxon signed-rank tests were conducted to compare the expression levels of NRP1 and ACE2 within the same samples, stratified by group (control or COVID-19). Differences were considered statistically significant at a *p*-value < 0.05.

## 3. Results

### 3.1. Conservation and Expression Analysis of NRP1

#### 3.1.1. Conservation of the Viral Binding Domain in SARS-CoV-2 Isolated in Mexico

Table 1 summarizes the conservation of the RRAR-binding domain within SARS-CoV-2 genomes isolated in Mexico between 2020 and 2024, along with associated lethality indices and confirmed case numbers. Across all years analyzed, the domain showed remarkably high conservation, consistently exceeding 98%. The lowest conservation was observed in 2020 (98.56%), while the highest was recorded in 2021 (99.33%), coinciding with the introduction of COVID-19 vaccines in Mexico. Conservation levels remained stable in subsequent years: 99.30% in 2022, 99.28% in 2023, and 99.11% in the most recent data from 2024 onwards.

The lethality index showed a marked downward trend over time. In 2020, the index was highest (9.81), reflecting the early impact of the pandemic. This value dropped significantly in 2021 (6.10) and reached its lowest in 2023 (0.78), likely influenced by increasing vaccine coverage and improved clinical management. Confirmed case numbers peaked in 2022 (3,219,032), while 2023 showed a notable decrease (355,424 as of June 24). No lethality or case data were available for 2024 at the time of analysis. All epidemiological data were obtained from the official Mexican COVID-19 database provided by CONACYT (https://datos.covid-19.conacyt.mx/#DownZCSV (accessed on 4 July 2025)) and analyzed using Microsoft Excel.

These results highlight both the genetic stability of the RRAR domain and the epidemiological shift in COVID-19 severity in Mexico across the years, supporting the domain’s relevance as a conserved therapeutic target.

#### 3.1.2. Conservation Analysis of the SARS-CoV-2–ACE2 Interaction Region

In parallel, the conservation of the spike protein region encoding the amino acids involved in binding to ACE2 (L455, F486, Q493, S494, and N501) was assessed.

The analyzed nucleotide region was as follows:

TTG TTT AGG AAG TCT AAT CTC AAA CCT TTT GAG AGA GAT ATT TCA ACT GAA ATC TAT CAG GCC GGT AGC ACA CCT TGT AAT GGT GTT GAA GGT TTT AAT TGT TAC TTT CCT TTA CAA TCA TAT GGT TTC CAA CCC ACT AAT

Table 2 presents the year-by-year conservation analysis of the spike protein region responsible for binding to ACE2, based on key residues (L455, F486, Q493, S494, and N501) encoded within the specified nucleotide sequence. In 2020, 88.09% of the sequences retained the reference binding region, but this conservation dropped drastically in subsequent years.

In 2021, only 6.15% of viral genomes maintained this region unchanged. By 2022, conservation had nearly disappeared (0.022%), and in 2023 no conserved sequences were detected (0%). A slight reappearance of the region was noted in 2024 (0.62%), though with negligible prevalence. This sharp decline in ACE2-binding region conservation over time stands in contrast to the stable conservation observed for the RRAR domain (see Table 1), suggesting stronger evolutionary pressure on the ACE2 interface, possibly due to immune evasion or vaccine-driven selection.

These findings emphasize the genetic instability of the ACE2-binding interface, raising challenges for long-term therapeutic strategies targeting ACE2 and reinforcing the importance of focusing on more conserved regions like the RRAR motif.

### 3.2. Expression Analysis of NRP1 and ACE2 in Lung Tissues

Table 3 presents transcriptomic expression data (TPM) for NRP1 and ACE2 in lung tissue samples from COVID-19 patients and healthy controls, based on publicly available RNA-Seq data (GSE150316). A total of 11 samples were included: 5 control lung tissues (3 males, 2 females; average age ~55 years) and 6 post-mortem COVID-19 lung tissues (4 males, 2 females; average age ~58 years). All COVID-19 cases corresponded to patients who died from severe disease, according to dataset annotations.

**Figure 1:**
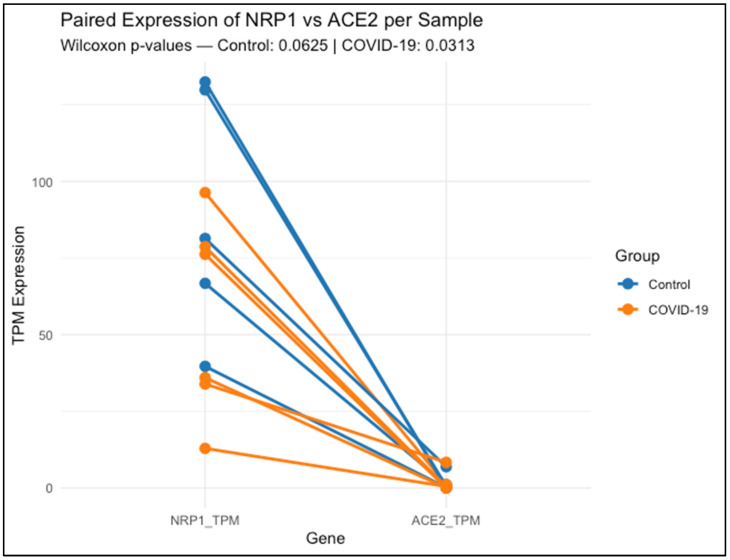
Paired expression of NRP1 and ACE2 in lung tissue from control and COVID-19 samples.

Figure 1 shows that each line connects the expression levels of NRP1 and ACE2 within the same lung tissue sample, either from healthy controls (blue) or COVID-19 patients (orange). 

Data represent transcript abundance in transcripts per million (TPM), as obtained from publicly available RNA-Seq data (GSE150316). Across nearly all samples, NRP1 expression was higher than ACE2, regardless of clinical group. Statistical comparison using the Wilcoxon signed-rank test revealed a non-significant trend in the control group (*p* = 0.0625), and a significant difference in the COVID-19 group (*p* = 0.03125), indicating that NRP1 expression remains elevated relative to ACE2 even during infection.

NRP1 expression was clearly higher and more consistent among control lung samples, ranging from 39.67 to 132.40 TPM. COVID-19 samples exhibited greater variability (range: 12.91 to 96.30 TPM), suggesting inter-individual differences in NRP1 regulation during infection. A paired Wilcoxon test comparing NRP1 and ACE2 expression within each sample showed a non-significant trend in controls (*p* = 0.0625), while a statistically significant difference was observed in COVID-19 patients (*p* = 0.03125), with NRP1 consistently exceeding ACE2 levels.

By contrast, ACE2 expression was low overall, with a heterogeneous and sporadic distribution across both groups. Several samples—both controls and COVID-19—displayed undetectable levels (0.000 TPM), while isolated cases reached moderate to high values (e.g., 6.927 TPM in one control and 8.355 TPM in a COVID-19 sample). This distribution highlights the limited and variable expression of ACE2 in lung tissue compared to the broader and more stable profile of NRP1.

### 3.3. Design and Cross-Species Conservation of siRNAs

Table 4 summarizes the coordinates of the coding region sequences (CDS) for multiple human NRP1 mRNA variants and the corresponding region in *Rattus norvegicus*, based on NCBI reference transcripts. In human transcripts, most variants (1–5 and 7) share a common translation start site at position 286, with coding region endpoints ranging between 2105 and 3057, depending on transcript length. Variant 6 was identified as non-coding, and was excluded from siRNA targeting. The rat NRP1 transcript exhibits a coding region extending from position 511 to 3295, slightly shifted relative to the human variants.

This comparative mapping confirmed that the 5′ region of the CDS is highly conserved across human variants and rat, supporting its use as a reliable target site for siRNA design. The siRNA target sequences were selected within this conserved region to ensure cross-species efficacy.

**Figure 2:**
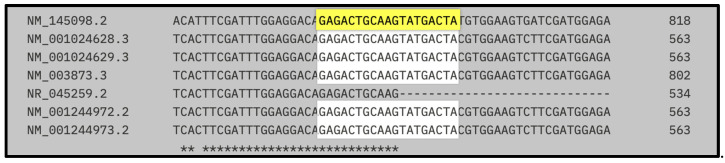
Localization of the first siRNA within human and rat NRP1 transcript variants.

Figure 2 illustrates the sequence alignment of the target site for the first designed siRNA, highlighting its position within the coding region of NRP1 mRNA across multiple human transcript variants and the rat (*Rattus norvegicus*) reference transcript (NM_145098.2).

The siRNA-binding site, shown in yellow, corresponds to the 19-nucleotide sequence GAGACTGCAAGTATGACTA, and is located within a highly conserved region present in all human coding variants assessed (NM_001024628.3, NM_001024629.3, NM_003873.3, NM_001244972.2, and NM_001244973.2), with exact sequence identity. Asterisks below the alignment indicate positions that are conserved across all sequences shown.

Table 5 presents the hybridization coordinates of the first designed siRNA (GAGACTGCAAGTATGACTA) within the mRNA transcripts of *Rattus norvegicus* and multiple human NRP1 variants. The siRNA aligns with a fully conserved region in both species, highlighting its suitability for cross-species silencing applications.

In all human variants analyzed (1–5 and 7), the siRNA-binding site is located between nucleotides 524 and 542, showing perfect sequence identity. In the rat transcript (NM_145098.2), the target region maps to positions 777–797, also exhibiting complete conservation of the 19-nt sequence.

These conserved coordinates confirm that the designed siRNA hybridizes within the coding region of NRP1 in all relevant isoforms and across species, providing strong support for its broad applicability in preclinical models and its potential translational relevance in therapeutic silencing strategies targeting NRP1.

**Figure 3:**
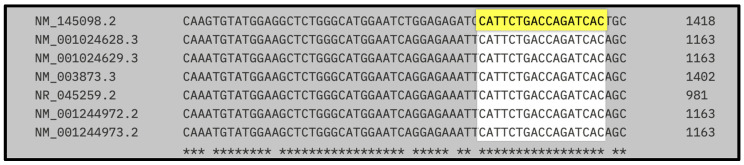
Localization of the second siRNA within human and rat NRP1 transcript variants.

Figure 3 shows the multiple sequence alignment of the binding site for the second siRNA candidate, highlighting its position across human and rat NRP1 mRNA transcript variants. The target sequence CATTCTGACCAGATCAC (highlighted in yellow) aligns perfectly across all human coding variants (including NM_001024628.3, NM_001024629.3, NM_003873.3, NM_001244972.2, and NM_001244973.2), as well as the rat reference transcript (NM_145098.2). Asterisks below the alignment indicate positions that are conserved across all sequences shown.

Table 6 provides the hybridization coordinates of the second siRNA designed against NRP1, targeting the 17-nucleotide sequence CATTCTGACCAGATCAC. This sequence was selected for its high conservation across species and was confirmed to be present without mismatches in all analyzed human NRP1 variants (1–5, 7), as well as in the rat transcript NM_145098.2.

In human transcripts, the siRNA target site consistently maps to positions 1144–1160 nt, while in *Rattus norvegicus*, it aligns between 1399–1415 nt, reflecting species-specific differences in untranslated regions but full conservation of the coding region itself.

These findings confirm that both siRNA-1 and siRNA-2 bind to structurally favorable, exposed regions of the transcript, supporting effective and reproducible gene silencing across species. This structural validation complements the sequence conservation data and reinforces the rationale for selecting these sites in the design of robust siRNA-based therapies targeting NRP1.

Table 7 summarizes the physicochemical characteristics of the siRNA-1 duplex used in this study. The duplex consists of a 21-nucleotide sense strand (5′-GAG ACT GCA AGT ATG ACT A-3′) and its complementary antisense strand (5′-TAG TCA TAC TTG CAG TCT CTT-3′), designed to target a conserved region of the NRP1 coding sequence.

The duplex displays a GC content of 38.1%, which favors moderate thermodynamic stability while minimizing off-target interactions. The melting temperature (Tm) was calculated at 61.3 °C, indicating a stable duplex formation under physiological conditions. The molecular weight of the duplex is 6362.2 g/mol, with an extinction coefficient of 193,600 L/(mol·cm), enabling precise quantification by spectrophotometry.

For optical density measurements at 260 nm, the siRNA has a conversion factor of 5.17 nmol per OD260 and 32.86 μg per OD260, which facilitates accurate dosing in experimental applications.

Details the physicochemical parameters of the second siRNA duplex designed to target NRP1. The duplex consists of a 19-nucleotide sense strand (5′-CATTCTGACCAGATCAC-3′) and a complementary antisense strand (5′-GTGATCTGGTCAGAATGTT-3′). Its shorter length compared to siRNA-1 contributes to enhanced target specificity and potentially reduced off-target effects (Table 8).

The GC content of 42.1% provides balanced thermodynamic stability, while the melting temperature (Tm) of 55.4 °C supports effective hybridization under physiological conditions. The calculated molecular weight is 5873.9 g/mol, and the extinction coefficient is 186,500 L/(mol·cm), enabling precise quantification by UV absorbance. For OD260-based quantification, this duplex yields 5.36 nmol and 31.5 µg per unit, which facilitates accurate preparation for experimental dosing.

### 3.4. Evaluation of Relative NRP1 mRNA Expression in Lung Tissue

The relative expression of NRP1 mRNA in lung tissue was evaluated by RT-PCR in rats, aiming to confirm its pulmonary expression. Primers were designed using the Roche ProbeFinder tool based on the reference sequence NM_145098.2 (*Rattus norvegicus* neuropilin-1 mRNA), and were synthesized by the Oligo T4 laboratory.

Two primer sets were used targeting distinct regions of NRP1, both optimized for real-time PCR and designed using the Universal ProbeLibrary system (Roche). Primers designed for detection of NRP1 in *Rattus norvegicus* are shown in Table 9. 

This table lists the primer sequences used for the quantification of NRP1 mRNA expression by RT-qPCR, along with two reference genes, β-actin (ACTB) and hypoxanthine-guanine phosphoribosyltransferase (HPRT), selected for normalization purposes.

Importantly, the use of two independent housekeeping genes—β-actin and HPRT-1—for normalization enhances the robustness and reliability of the findings. This dual-normalization approach minimizes potential reference gene bias and supports the reproducibility and consistency of the observed NRP1 downregulation.

### 3.5. Physiological Parameters Following siRNA Administration

Table 10, Table 11 and Table 12 summarize the systolic arterial pressure (SAP), diastolic arterial pressure (DAP), and heart rate (HR) measured at baseline (time zero) and three days after siRNA administration across all experimental groups: Control, Sham, Control + Vehicle, and siRNA + Vehicle.

At baseline, all groups showed comparable SAP, DAP, and HR values. Notably, the siRNA + Vehicle group displayed physiological parameters within normal ranges, similar to those of the control groups, indicating that systemic administration of the siRNA formulation did not induce any acute cardiovascular alterations.

Three days post-injection, all hemodynamic parameters remained stable, with no significant differences among the groups. This reinforces the hemodynamic safety profile of the siRNA compound, even after systemic exposure and potential intracellular uptake.

In addition, Table 12 presents the body weight measurements recorded at time zero and three days post-treatment. All groups showed a slight increase in weight, proportional to normal growth patterns expected during the experimental period. No statistically significant differences in weight gain were observed, further supporting the absence of systemic toxicity or adverse metabolic effects associated with the siRNA treatment.

### 3.6. Evaluation of the Effect of siRNA Administration in a Melittin-Induced Lung Injury Model

To determine the effect of the designed siRNAs, lung damage was assessed in a melittin-induced injury model in both the control group (vehicle) and the siRNA-treated group. ImageJ software was used to quantify the total lung area, lesion area, and percentage of affected (injured) tissue.

The protective effect observed in the Melittin + siRNA group was achieved by administering 50 µL of siRNA formulation intravenously via the jugular vein, 30 min prior to melittin exposure. This timing was selected to allow for sufficient intracellular uptake before the onset of inflammatory injury.

The administered siRNAs exerted a protective effect on lung tissue, as the extent of injury following melittin administration was significantly lower in the siRNA-treated groups compared to the control group.

**Figure 4:**
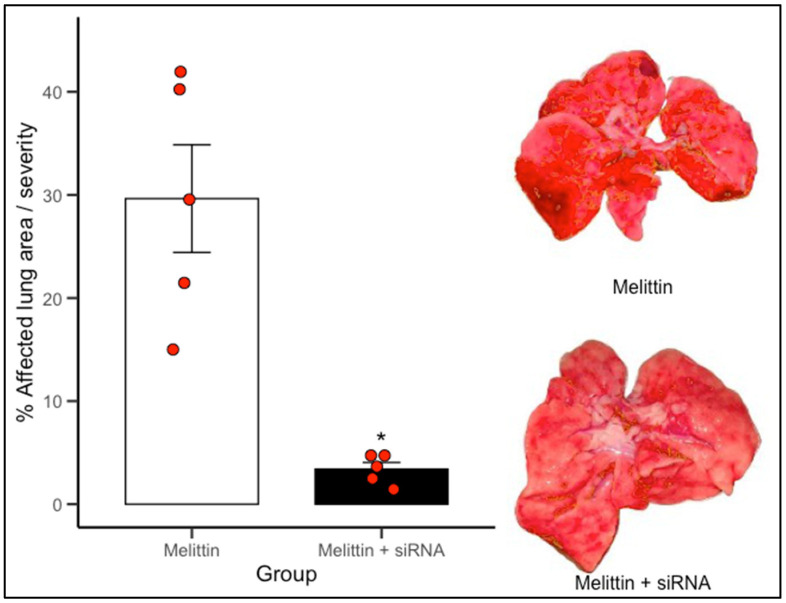
siRNA targeting NRP1 reduces melittin-induced lung injury.

The Figure 4 illustrates the extent of macroscopic lung damage following melittin-induced inflammation, quantified as the percentage of visibly affected pulmonary surface. Lungs from animals treated only with melittin displayed extensive tissue injury, with mean lesion areas approximating 30%, characteristic of acute inflammatory insult. In contrast, animals pretreated with siRNA 1–2 directed against NRP1 showed a notable reduction in pulmonary damage, with lesion coverage falling to below 10% on average. This difference was statistically significant (*p* < 0.05). The visual comparison between groups, supported by representative lung images, underscores the protective effect of NRP1 knockdown. * Indicate statistically significant differences (*p* < 0.05) versus Melittin.

## 4. Discussion

This study provides a comprehensive evaluation of the therapeutic potential of small interfering RNAs (siRNAs) targeting neuropilin-1 (NRP1), a multifunctional host co-receptor implicated in SARS-CoV-2 cellular entry and inflammatory signaling. Our integrative approach combined transcriptomic profiling of human lung tissue, rational siRNA design validated through interspecies sequence conservation, and in vivo functional assessment using a chemically induced model of acute lung injury.

In this model, melittin—the principal cytolytic peptide in bee venom—was used as a potent inducer of lung damage. Melittin acts via multiple mechanisms, including disruption of cellular membranes, activation of phospholipase A2, and interaction with cell surface receptors such as NRP1, which mediates its internalization via endocytosis. Once internalized, melittin promotes mitochondrial depolarization, reactive oxygen species (ROS) generation, and downstream apoptotic signaling cascades, contributing to its cytotoxic effects [10,11].

This injury model allowed us to evaluate both the biological impact and physiological consequences of NRP1 knockdown in a pathologically relevant setting. Furthermore, longitudinal sequence analyses of SARS-CoV-2 spike variants confirmed strong conservation of the RRAR motif—the canonical NRP1-binding site—across recent lineages. These data, together with differential expression patterns of host receptors, further support the prioritization of NRP1 as a gene-silencing target. Collectively, our findings underscore the translational potential of NRP1-targeted RNAi strategies to modulate viral infectivity and host response [10,11].

We found that the RRAR furin cleavage motif in the SARS-CoV-2 spike protein maintained >98% conservation across viral isolates from Mexico between 2020 and 2025, despite extensive genomic evolution. This polybasic site is essential for spike activation and enables high-affinity binding to NRP1’s b1 domain. (Table 1) [1,2,3,4,12].

In contrast, the ACE2-binding region in the receptor-binding domain (RBD) showed dramatic loss of conservation (Table 2). This divergence likely reflects strong antibody-driven selection pressure, favoring RBD mutations that enable immune escape. These findings support previous studies showing that variants of concern (e.g., Delta, Omicron) accumulate mutations in the RBD to increase ACE2 affinity or evade neutralizing antibodies [13]. Therefore, the spike–NRP1 axis represents a more conserved and stable viral–host interface than spike–ACE2.

To complement these findings, we analyzed NRP1 and ACE2 transcript levels in lung samples from healthy individuals and COVID-19 patients using data from GEO (GSE150316). NRP1 showed higher and more consistent expression in controls, while expression in COVID-19 patients, although reduced, remained detectable (Figure 1). In contrast, ACE2 levels were highly variable and often undetectable, echoing previous reports (Table 3) [14]. These results further emphasize NRP1’s biological robustness and reinforce its candidacy as a therapeutic target [3,13,14,15].

Taken together, these findings confirm that the spike–NRP1 interface combines evolutionary conservation and stable tissue expression, in contrast to the mutable spike–ACE2 interaction. This stability makes NRP1 an attractive host-directed target in antiviral strategies.

To improve translational relevance, we designed two siRNA candidates based on conserved regions of NRP1 mRNA shared between *Homo sapiens* and *Rattus norvegicus*. Alignment using Clustal Omega [16] revealed perfect conservation of the selected target regions across five human transcript variants and the rat ortholog (Table 4) [17].

Predicted RNA secondary structures, analyzed via RNAfold, confirmed that both siRNA-1 and siRNA-2 target sites localized to accessible regions—stem loops or unstructured motifs—known to favor RISC complex binding (Figure 2 and Figure 3). siRNA-1 (5′-GAGACTGCAAGTATGACTA-3′) consistently mapped to accessible loop domains, while siRNA-2 (5′-CATTCTGACCAGATCAC-3′) was located in extended unstructured regions. These features, along with favorable melting temperatures and extinction coefficients, indicate high silencing potential and quantification reliability (Table 5 and Table 6).

**Figure 5:**
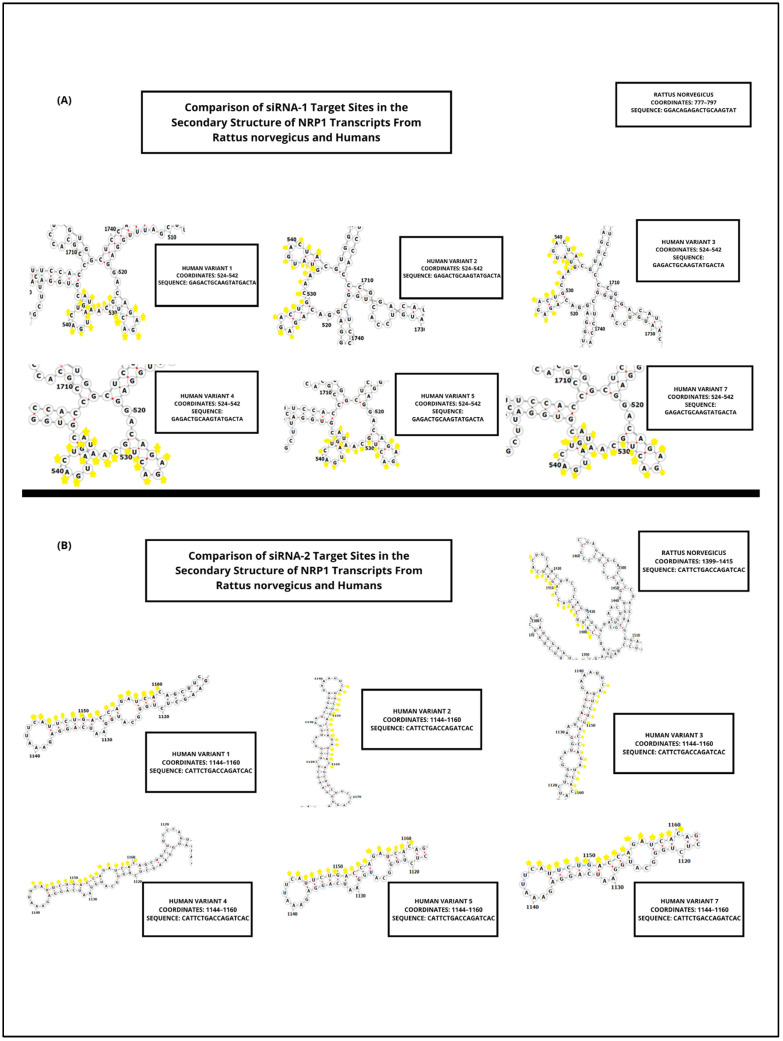
Predicted secondary structures of NRP1 mRNA showing siRNA target site accessibility (RNAfold analysis). (**A**) Secondary structure predictions of NRP1 mRNA transcripts from *Rattus norvegicus* and multiple human variants (1–5 and 7), highlighting the hybridization site of siRNA-1 (sequence: GAGACTGCAAGTATGACTA) in yellow. The target region consistently localizes within accessible loop or stem-loop structures across all transcripts analyzed, favoring efficient RISC complex binding and RNA interference. These structures were generated using the RNAfold WebServer, which estimates the minimum free energy folding conformation of RNA sequences. (**B**) Predicted secondary structures of the same NRP1 mRNA variants, showing the hybridization site of siRNA-2 (sequence: CATTCTGACCAGATCAC) in yellow. Similar to siRNA-1, this target site is consistently positioned within loop or stem-loop regions that enhance accessibility for RNA-induced silencing.

Structural accessibility was further supported by comparative secondary structure analyses across human and rat transcripts (Figure 5).

Both siRNAs targeted regions with consistent unstructured motifs, even across minor transcript variations. This supports the stability of target site accessibility—a critical determinant for effective gene silencing [18].

These findings are consistent with previous reports [18], who demonstrated that local RNA folding better reflects in vivo accessibility than global structure models. Their LocalFold algorithm prioritizes short-range interactions and accounts for edge effects, offering a robust framework for target accessibility prediction. Our results confirm this principle, as siRNA-binding sites retained accessibility despite local variations in folding [18].

This multi-tiered validation—combining interspecies conservation, structural accessibility, and physicochemical profiling—supports the use of our siRNAs in both preclinical and translational settings.

**Figure 6:**
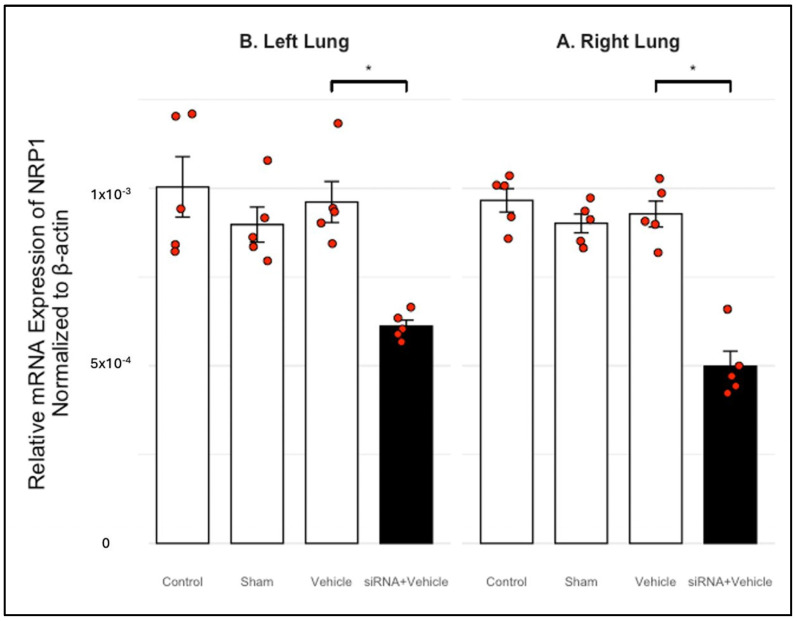
siRNA 1–2 mediated knockdown of NRP1 in rat lung tissue (normalized to β-actin). (**A**) Relative mRNA expression levels of NRP1 in the right lung across four experimental groups: Control, Sham, Vehicle, and siRNA + Vehicle. No significant differences were observed among the Control, Sham, and Vehicle groups, indicating that the surgical procedure and vehicle formulation did not alter baseline NRP1 expression. In contrast, the siRNA + Vehicle group exhibited a significant reduction in NRP1 expression (*p* < 0.05), confirming the effectiveness of siRNA-mediated gene silencing. (**B**) Same analysis conducted in the left lung. The results mirrored those observed in the right lung, with comparable expression among the Control, Sham, and Vehicle groups, and a significant reduction in the siRNA-treated group (*p* < 0.05). These findings demonstrate a consistent and bilateral knockdown effect and validate the efficiency of systemic siRNA delivery for targeting NRP1 in pulmonary tissue. * Indicate statistically significant differences (*p* < 0.05) versus Control.

**Figure 7:**
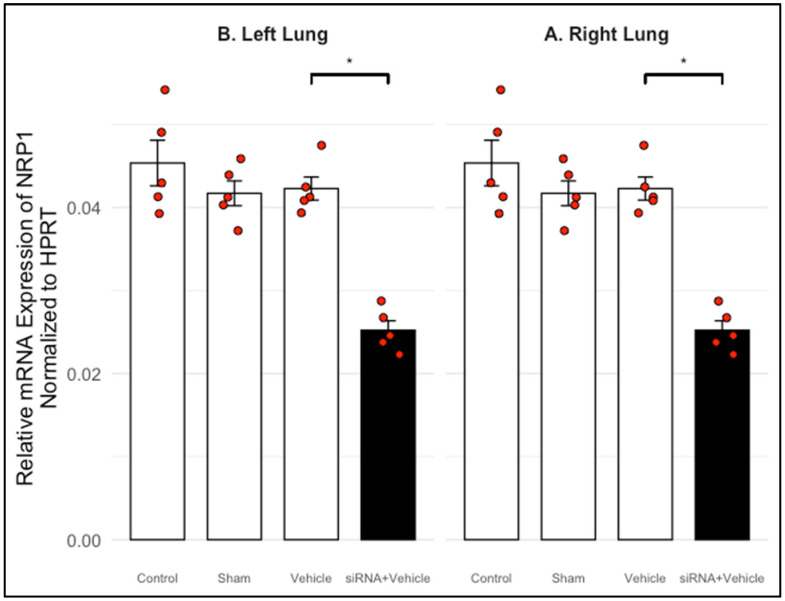
siRNA 1–2 mediated knockdown of NRP1 in rat lung tissue (normalized to HPRT-1). (**A**) Relative mRNA expression levels of NRP1 in the right lung, using HPRT-1 as the reference gene. The Control, Sham, and Vehicle groups displayed comparable expression levels, indicating that the injection procedure and vehicle formulation did not affect basal NRP1 expression. In contrast, the siRNA + Vehicle group exhibited a significant reduction in NRP1 expression (*p* < 0.05), confirming the efficacy of the siRNA-mediated knockdown. (**B**) Relative mRNA expression levels of NRP1 in the left lung, also normalized to HPRT-1. As in the right lung, the siRNA + Vehicle group showed a significant decrease compared to all other groups (*p* < 0.05), while Control, Sham, and Vehicle groups remained statistically similar. These results reinforce the bilateral effect of siRNA treatment and the robustness of the knockdown across lung lobes. * Indicate statistically significant differences (*p* < 0.05) versus Control.

To suggest in vivo efficacy, we quantified NRP1 mRNA levels in rat lung tissue after intravenous administration of siRNA via the jugular vein. Quantitative RT-PCR revealed a significant reduction in NRP1 transcript levels in the right lung of treated animals versus controls. A similar reduction was observed in the left lung, indicating bilateral silencing efficacy (Table 9, Figure 6 and Figure 7).

To evaluate safety, we monitored systolic/diastolic blood pressure, heart rate, and body weight at baseline and three days post-treatment. No significant alterations were detected, and all values remained within physiological reference ranges (Table 10, Table 11 and Table 12). These findings confirm that the siRNA formulations were well-tolerated and did not induce cardiovascular or metabolic toxicity.

Image evaluation of melittin-induced lung injury showed marked reduction in tissue damage among siRNA-treated animals (Figure 7). This suggests that NRP1 may contribute to inflammation and tissue injury via mechanisms involving vascular permeability, immune cell recruitment, or cytokine signaling [1,10,11].

Collectively, our results demonstrate that NRP1 silencing not only reduces transcript levels but also confers functional protection in acute lung injury. These data validate NRP1 as a modifiable host factor and support siRNA-based therapeutic strategies targeting this axis.

## 5. Conclusions

This study provides compelling evidence supporting neuropilin-1 (NRP1) as a viable and translationally relevant therapeutic target for gene-silencing strategies against SARS-CoV-2 infection and chemically induced lung injury. Through a multidisciplinary approach—encompassing viral sequence conservation analysis, pulmonary transcriptomic profiling, rational siRNA design, and in vivo validation—we demonstrated the biological and therapeutic value of targeting the spike–NRP1 interaction.

The RRAR furin cleavage site of the SARS-CoV-2 spike protein, which directly engages with the b1 domain of NRP1, remained highly conserved in viral isolates from Mexico over five consecutive years. In parallel, NRP1 expression in human lung tissue was consistently detectable and less variable than ACE2, highlighting its biological robustness and reliability as a host factor. These dual attributes—sequence conservation and stable tissue expression—position NRP1 as a strategic target less vulnerable to immune-driven viral evolution.

Two siRNA constructs were designed to specifically silence conserved regions of NRP1 mRNA shared between humans and rodents. Both sequences exhibited favorable thermodynamic properties, structural accessibility, and cross-species compatibility, ensuring their functional applicability in translational models. In vivo administration of the siRNAs achieved significant bilateral NRP1 knockdown in rat lung tissue without eliciting systemic toxicity, as evidenced by stable physiological parameters. Importantly, NRP1 silencing markedly reduced pulmonary damage in a melittin-induced lung injury model, suggesting a role for NRP1 in modulating inflammatory or cytotoxic responses.

Together, these findings highlight the potential of siRNA-based targeting of NRP1 not only in the context of SARS-CoV-2, but also in broader inflammatory and vascular pulmonary disorders. NRP1 has been shown to mediate inflammation by promoting vascular permeability, leukocyte transmigration, and the amplification of pro-inflammatory signaling cascades through interactions with ligands such as VEGF-A and semaphorins. Its role as a modulator of immune cell recruitment and cytokine release further underscores its relevance as a therapeutic target in acute and chronic lung injury. Future studies focused on delivery optimization, long-term efficacy, and testing in advanced disease models will be essential to transition this therapeutic concept toward clinical application.

## Figures and Tables

**Table 1:** Conservation of the RRAR-binding domain in SARS-CoV-2 genomes isolated in Mexico.

Year	Sequences Analyzed	Sequences with Domain	% Conservation	Lethality Index	Confirmed Cases
2020	3760	3706	98.56%	9.81	1,522,878
2021	50,886	50,546	99.33%	6.10 (Arrival of vaccine in Mexico, February 2021)	2,536,021
2022	37,436	37,175	99.30%	0.86	3,219,032
2023	7623	7568	99.28%	0.78	355,424 (Data as of 24 June 2023)
2024 onwards	5032	4987	99.11%	-	-

**Table 2:** Conservation of the ACE2-binding region in SARS-CoV-2 genomes isolated in Mexico.

Year	Sequences Analyzed	Sequences with Region Union	% Conservation	Lethality Index	Confirmed Cases
2020	3760	3312	88.09	9.81	1,522,878
2021	50,886	3416	6.15	6.10	2,536,021
2022	37,436	8	0.022	0.86	3,230,329
2023	7623	0	0	0.78	355,424 (Data as of 24 June 2023)
2024 onwards	5032	31	0.62	-	-

**Table 3:** Comparative expression analysis of NRP1 and ACE2 in lung tissue from COVID-19 patients and controls.

Group	Sample	NRP1 (TPM)	ACE2 (TPM)
Control	GSM4546608	129.80	0.568
Control	GSM4546609	39.67	0.133
Control	GSM4546610	132.40	0.000
Control	GSM4546611	66.73	1.114
Control	GSM4546612	81.35	6.927
COVID-19	GSM4546576	96.30	1.087
COVID-19	GSM4546596	12.91	0.496
COVID-19	GSM4698526	78.66	0.413
COVID-19	GSM4698536	33.91	8.355
COVID-19	GSM4698544	35.97	0.000
COVID-19	GSM4698549	76.21	0.000

**Table 4:** Coding region coordinates of NRP1 mRNA in human and *Rattus norvegicus* variants.

Species/Variant	Coding Region Coordinates
Human variant 1	286–3057
Human variant 2	286–2160
Human variant 3	286–2105
Human variant 4	286–3039
Human variant 5	286–3036
Human variant 6	Non-coding
Human variant 7	286–3006
*Rattus norvegicus*	511–3295

**Table 5:** Conserved hybridization coordinates of siRNA-1 across rat and human NRP1 variants.

Species/Variant	siRNA-1 Sequence	Hybridization Coordinates (nt)
*Rattus norvegicus*	GAGACTGCAAGTATGACTA	777–797
Human variant 1	GAGACTGCAAGTATGACTA	524–542
Human variant 2	GAGACTGCAAGTATGACTA	524–542
Human variant 3	GAGACTGCAAGTATGACTA	524–542
Human variant 4	GAGACTGCAAGTATGACTA	524–542
Human variant 5	GAGACTGCAAGTATGACTA	524–542
Human variant 7	GAGACTGCAAGTATGACTA	524–542

**Table 6:** Conserved hybridization coordinates of siRNA-2 across rat and human NRP1 variants.

Species/Variant	siRNA-2 Sequence	Hybridization Coordinates (nt)
*Rattus norvegicus*	CATTCTGACCAGATCAC	1399–1415
Human variant 1	CATTCTGACCAGATCAC	1144–1160
Human variant 2	CATTCTGACCAGATCAC	1144–1160
Human variant 3	CATTCTGACCAGATCAC	1144–1160
Human variant 4	CATTCTGACCAGATCAC	1144–1160
Human variant 5	CATTCTGACCAGATCAC	1144–1160
Human variant 7	CATTCTGACCAGATCAC	1144–1160

**Table 7:** Physicochemical properties of siRNA-1 duplex.

Property	Value
Sense strand (5′→3′)	GAG ACT GCA AGT ATG ACT A
Antisense strand (5′→3′)	TAG TCA TAC TTG CAG TCT CTT
Length	21 nucleotides
GC content	38.1%
Melting temperature (Tm)	61.3 °C
Molecular weight	6362.2 g/mol
Extinction coefficient	193,600 L/(mol·cm)
nmol per OD260	5.17 nmol
µg per OD260	32.86 µg

**Table 8:** Physicochemical properties of siRNA-2 duplex.

Property	Value
Sense strand (5′→3′)	CATTCTGACCAGATCAC
Antisense strand (5′→3′)	GTGATCTGGTCAGAATGTT
Length	19 nucleotides
GC content	42.1%
Melting temperature (Tm)	55.4 °C
Molecular weight	5873.9 g/mol
Extinction coefficient	186,500 L/(mol·cm)
nmol per OD260	5.36 nmol
µg per OD260	31.5 µg

**Table 9:** Primer characteristics for NRP1 detection.

Primer	Sequence (5′→3′)
**NRP1 Forward**	ACGAGTGTGACGATGACCAG
**NRP1 Reverse**	CCTCCTGTGAGCTGGAAGTC
**ACTB Forward**	CGTCATCCATGGCGAACT
**ACTB Reverse**	CCCGCGAGTACAACCTTCT
**HPRT Forward**	CAATCAAGACGTTCTTTCCAGTT
**HPRT Reverse**	GCTCCATTCCTATGACTGTAGATTTT

**Table 10:** Systolic arterial pressure (SAP), diastolic arterial pressure (DAP), and heart rate (HR) at time zero.

Parameter	C. Control	Sham	C + Vehicle	siRNA + Vehicle
SAP (mmHg)	107.80 ± 1.20	115.50 ± 2.24	116.90 ± 0.98	108.06 ± 1.03
DAP (mmHg)	77.40 ± 1.00	77.07 ± 1.38	78.60 ± 0.55	76.80 ± 1.28
HR (beats/min)	388.50 ± 5.40	385.50 ± 3.59	381.30 ± 1.46	384.24 ± 3.62

**Table 11:** Systolic arterial pressure (SAP), diastolic arterial pressure (DAP), and heart rate (HR) three days after siRNA administration.

Parameter	C. Control	Sham	C + Vehicle	siRNA + Vehicle
SAP (mmHg)	106.60 ± 1.20	112.89 ± 2.24	112.20 ± 1.58	106.40 ± 1.03
DAP (mmHg)	77.00 ± 1.00	75.47 ± 1.38	77.40 ± 0.87	76.66 ± 1.28
HR (beats/min)	382.00 ± 5.40	384.60 ± 3.59	383.13 ± 3.64	386.20 ± 3.62

**Table 12:** Body weight (in grams) at time zero and three days post-siRNA administration.

Time Point	C. Control	Sham	C + Vehicle	siRNA + Vehicle
Time zero	345.70 ± 4.20	348.90 ± 6.24	359.30 ± 2.98	358.60 ± 3.03
Day three post-siRNA	355.70 ± 7.00	358.02 ± 6.83	361.20 ± 9.55	361.13 ± 9.34

## Data Availability

The original data used and analyzed in this study are available in public databases. SARS-CoV-2 sequences were obtained from GISAID (https://www.gisaid.org/). Gene expression data were obtained from the GEO repository under accession number GSE150316 (https://www.ncbi.nlm.nih.gov/geo/query/acc.cgi?acc=GSE150316 (accessed on 4 July 2025)). Additionally, COVID-19 data from the Mexican national CONACYT repository (https://datos.covid-19.conacyt.mx/#DownZCSV (accessed on 4 July 2025)) were used. All additional data generated or analyzed during this study are included in this published article.

## References

[1] Nguyen H.L., Hieu H.K., Nguyen T.Q., Nhung N.T.A., Li M.S. (2024). Neuropilin-1 Protein May Serve as a Receptor for SARS-CoV-2 Infection: Evidence from Molecular Dynamics Simulations. J. Phys. Chem. B.

[2] Daly J.L., Simonetti B., Klein K., Chen K.-E., Williamson M.K., Antón-Plágaro C., Shoemark D.K., Simón-Gracia L., Bauer M., Hollandi R. (2020). Neuropilin-1 Is a Host Factor for SARS-CoV-2 Infection. Science.

[3] Cantuti-Castelvetri L., Ojha R., Pedro L.D., Djannatian M., Franz J., Kuivanen S., van der Meer F., Kallio K., Kaya T., Anastasina M. (2020). Neuropilin-1 Facilitates SARS-CoV-2 Cell Entry and Infectivity. Science.

[4] Hoffmann M., Kleine-Weber H., Pöhlmann S. (2020). A Multibasic Cleavage Site in the Spike Protein of SARS-CoV-2 Is Essential for Infection of Human Lung Cells. Mol. Cell.

[5] Hou D., Cao W., Kim S., Cui X., Ziarnik M., Im W., Zhang X.F. (2023). Biophysical Investigation of Interactions between SARS-CoV-2 Spike Protein and Neuropilin-1. Protein Sci..

[6] Dougherty D.A. (2013). The Cation−π Interaction. Acc. Chem. Res..

[7] Zabidi N.Z., Liew H.L., Farouk I.A., Puniyamurti A., Yip A.J.W., Wijesinghe V.N., Low Z.Y., Tang J.W., Chow V.T.K., Lal S.K. (2023). Evolution of SARS-CoV-2 Variants: Implications on Immune Escape, Vaccination, Therapeutic and Diagnostic Strategies. Viruses.

[8] Zhao F., Zai X., Zhang Z., Xu J., Chen W. (2022). Challenges and Developments in Universal Vaccine Design against SARS-CoV-2 Variants. NPJ Vaccines.

[9] (2001). Especificaciones Técnicas Para la Producción, Cuidado y Uso de los Animales de Laboratorio.

[10] Nguyen C.D., Yoo J., Jeong S.J., Ha H.-A., Yang J.H., Lee G., Shin J.C., Kim J.-H. (2024). Melittin-the Main Component of Bee Venom: A Promising Therapeutic Agent for Neuroprotection through Keap1/Nrf2/HO-1 Pathway Activation. Chin. Med..

[11] Pandey P., Khan F., Khan M.A., Kumar R., Upadhyay T.K. (2023). An Updated Review Summarizing the Anticancer Efficacy of Melittin from Bee Venom in Several Models of Human Cancers. Nutrients.

[12] Lisewski A.M. (2024). Pre-Pandemic Artificial MERS Analog of Polyfunctional SARS-CoV-2 S1/S2 Furin Cleavage Site Domain Is Unique among Spike Proteins of Genus Betacoronavirus. BMC Genom. Data.

[13] Shang J., Ye G., Shi K., Wan Y., Luo C., Aihara H., Geng Q., Auerbach A., Li F. (2020). Structural Basis of Receptor Recognition by SARS-CoV-2. Nature.

[14] Malik J.R., Acharya A., Avedissian S.N., Byrareddy S.N., Fletcher C.V., Podany A.T., Dyavar S.R. (2023). ACE-2, TMPRSS2, and Neuropilin-1 Receptor Expression on Human Brain Astrocytes and Pericytes and SARS-CoV-2 Infection Kinetics. Int. J. Mol. Sci..

[15] Da Costa C.H.S., de Freitas C.A.B., Alves C.N., Lameira J. (2022). Assessment of Mutations on RBD in the Spike Protein of SARS-CoV-2 Alpha, Delta and Omicron Variants. Sci. Rep..

[16] Chenna R. (2003). Multiple Sequence Alignment with the Clustal Series of Programs. Nucleic Acids Res..

[17] Cabrera-Becerra S.E., Vera-Juárez G., García-Rubio V.G., Ocampo-Ortega S.A., Blancas-Napoles C.M., Aguilera-Méndez A., Romero-Nava R., Huang F., Hong E., Villafaña S. (2022). SiRNA Knockdown of Angiopoietin 2 Significantly Reduces Neovascularization in Diabetic Rats. J. Drug Target..

[18] Lange S.J., Maticzka D., Möhl M., Gagnon J.N., Brown C.M., Backofen R. (2012). Global or Local? Predicting Secondary Structure and Accessibility in MRNAs. Nucleic Acids Res..

